# Osteoclast-independent osteocyte dendrite defects in mice bearing the osteogenesis imperfecta-causing *Sp7 R342C* mutation

**DOI:** 10.1038/s41413-025-00440-1

**Published:** 2025-07-19

**Authors:** Jialiang S. Wang, Katelyn Strauss, Caroline Houghton, Numa Islam, Sung-Hee Yoon, Tatsuya Kobayashi, Daniel J. Brooks, Mary L. Bouxsein, Yingshe Zhao, Cristal S. Yee, Tamara N. Alliston, Marc N. Wein

**Affiliations:** 1 Charles and Jane Pak Center for Mineral Metabolism and Clinical Research, University of Texas Southwestern Medical Center, Dallas, TX USA; 2 Department of Physiology, University of Texas Southwestern Medical Center, Dallas, TX USA; 3 Department of Internal Medicine, University of Texas Southwestern Medical Center, Dallas, TX USA; 4 Endocrine Unit, Massachusetts General Hospital, Harvard Medical School, Boston, MA USA; 5 Department of Orthopedic Surgery, Center for Advanced Orthopedic Studies, Beth Israel Deaconess Medical Center, Harvard Medical School, Boston, MA USA; 6 Department of Orthopaedic Surgery, University of California in San Francisco, San Francisco, CA USA; 7 Broad Institute of Harvard and MIT, Cambridge, MA USA; 8 Harvard Stem Cell Institute, Cambridge, MA USA

**Keywords:** Bone, Endocrine system and metabolic diseases

## Abstract

Osteogenesis imperfecta (OI) is a group of diseases caused by defects in type I collagen processing which result in skeletal fragility. While these disorders have been regarded as defects in osteoblast function, the role of matrix-embedded osteocytes in OI pathogenesis remains largely unknown. Homozygous human *SP7* (*c.946* *C* > *T*, *R316C*) mutation results in a recessive form of OI characterized by fragility fractures, low bone mineral density and osteocyte dendrite defects. To better understand how the OI-causing *R316C* mutation affects the function of SP7, we generated *Sp7*^*R342C*^ knock-in mice. Consistent with patient phenotypes, *Sp7*^*R342C/R342C*^ mice demonstrate increased cortical porosity and reduced cortical bone mineral density. *Sp7*^*R342C/R342C*^ mice show osteocyte dendrite defects, increased osteocyte apoptosis, and intracortical bone remodeling with ectopic intracortical osteoclasts and elevated osteocyte *Tnfsf11* expression. Remarkably, these defects in osteocyte function contrast to only mild changes in mature osteoblast function, suggesting that this *Sp7* mutation selectively interferes with the function of Sp7 in osteocytes and mature osteoblasts, but not during early stages of osteoblast differentiation. Osteocyte morphology changes in *Sp7*^*R342C/R342C*^ mice were not restored by inhibiting osteoclast formation, indicating that dendrite defects lie upstream of high intracortical osteoclast activity in this model. Moreover, transcriptomic profiling reveals that the expression of a core set osteocyte-enriched genes is highly dysregulated by the *R342C* mutation. Thus, this supports a model in which osteocyte dysfunction can drive OI pathogenesis and provides a valuable resource to test novel therapeutic approaches and to understand the osteocyte-specific role of SP7 in bone remodeling.

## Introduction

Osteogenesis imperfecta (OI) is a collection of rare genetic bone disorders due to abnormal bone matrix and increased bone fragility. The majority of OI cases are caused by mutations in *COL1A1* and *COL2A1* (the main fibrillar collagens expressed in bone) and in other genes encoding proteins that control type I collagen biosynthesis by osteoblasts.^1,2^ Notably, many genes that are important for osteoblast formation have been identified to cause skeletal fragility and phenotypes similar to collagen-mutated OI.^3^ Osteocytes are the most abundant and longest-lived cells in bone. Surrounded by mineralized matrix, osteocytes possess an elaborate network of dendrite-like connections that are used for mechano-sensing and inter-cellular communication.^4^ Although morphological changes in osteocytes have been noted in rare instances in OI,^5–10^ the role of osteocytes in OI pathogenesis remains largely unexplored.

Osterix/Sp7 (encoded by the *Sp7* gene) is a zinc finger-containing transcription factor with an essential role in osteoblast differentiation.^11^ In *Sp7-*deficient pups, osteoblasts are absent, and there is no bone formation. Recent studies suggest distinct roles of Sp7 in chondrocytes and osteocytes.^12,13^ We deleted *Sp7* using *Dmp1-Cre*, which targets mature osteoblasts and osteocytes;^14^ these mutant mice showed increased cortical porosity, abnormal intracortical bone remodeling, osteocyte dendrite defects, and increased osteocyte apoptosis.^13^ Single-cell RNA sequencing identified subpopulations of cells undergoing the osteoblast-to-osteocyte transition, and dramatic osteocyte differentiation defects in the absence of Sp7. Overexpression of *Sp7* under the control of the 2.3 kb *Col1a1* promoter led to porous cortical bone, decreased number of osteocyte dendrites, disrupted lacunar-canalicular network and increased sclerostin expression upon skeletal unloading.^15^ Therefore, the osteocytes are quite sensitive to *Sp7* gene dosage. Overall, these findings raise the hypothesis that proper Sp7 expression and activity are required for osteocyte formation and bone development.

Rare *SP7* mutations in humans cause both recessive and dominant osteogenesis imperfecta (OI).^16–20^ Three siblings (two boys and one girl) with recessive pattern OI were found to have the homozygous *SP7* (*c.946* *C* > *T*) mutation.^16^ These patients presented with short stature, low-trauma fractures, low bone mineral density (BMD), mild scoliosis, hearing loss, facial dysmorphism, and delayed tooth eruption. Another recent study reported two male siblings with osteogenesis and dentinogenesis imperfecta from an Arabian family in Kuwait carrying the same mutation.^20^ This *SP7* mutation results in a substitution from arginine to cysteine (R316C). We analyzed osteocyte morphology in non-decalcified iliac crest bone biopsies from two teenage boys homozygous for the *R316C* mutation and two age- and sex-matched healthy controls and noted reduced osteocyte dendrite length and number in patients versus healthy controls.^13^ Importantly, such individuals showed preserved bone formation indices, suggesting that the *SP7 R316C* mutation may not affect the function of this transcription factor in osteoblasts.

To examine whether and how the OI-causing *R316C* mutation affects the function of SP7 in osteocytes, we generated *Sp7 R342C* mice (the arginine is located at position 342 in mice). Here we report that this genetically modified mouse strain mimics the bone phenotype observed in humans with this mutation. Both male and female mice with the *Sp7 R342C* mutation exhibit bone loss, increased cortical bone porosity, and osteocyte dendrite defects. Osteocyte dendrite defects are associated with increased osteocyte apoptosis, elevated *Tnfsf11* (gene encoding Rankl) expression, and increased osteoclast activity. However, osteocyte dendrite defects caused by the *R342C* mutation are not rescued when the osteoclast activity is inhibited, demonstrating that high bone resorption lies ‘downstream’ of osteocyte dendrite defects in this model. Bone transcriptome analysis in this model identified dysregulated expression of the osteocyte signature genes, findings in stark contrast to intact osteoblast function in cells bearing this mutation. In summary, this novel mouse model of osteogenesis imperfecta provides important insights into the role of osteocytes in OI. Furthermore, these findings support a model in which a single transcription factor can exert different functions during distinct stages of cell differentiation.

## Results

### Generation of *Sp7*^*R342C*^ knock-in mice

**Fig. 1:**
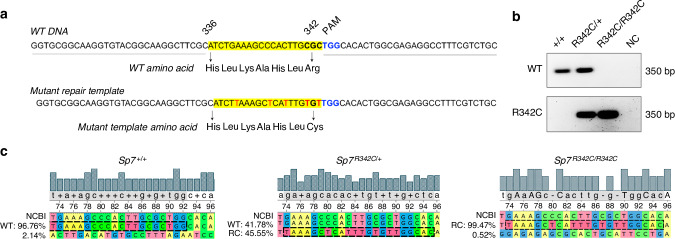
Generation of *Sp7*^*R342C*^ knock-in mice. **a** Wild-type mouse genomic DNA and mutant repair template sequences. Edited nucleotides are highlighted in red. 342 position (bold) is next to the PAM sequence (highlighted in blue). **b** Genotyping results of wild-type (*Sp7*^*+/+*^), heterozygous (*Sp7*^*R342C/+*^) and homozygous (*Sp7*^*R342C/R342C*^) mice. WT: wildtype primers; R342C: *R342C* mutant primers. NC: negative control with no DNA. **c** Next-generation sequencing of wild-type (*Sp7*^*+/+*^), heterozygous (*Sp7*^*R342C/+*^) and homozygous (*Sp7*^*R342C/R342C*^) mice. WT: sequence conservation to the wildtype allele; R342C: sequence conservation to the *R342C* mutant allele. Primer sequences are listed in Table S1

The *c.946* *C* > *T* mutation is located in exon 2 of human *SP7* gene and results in a substitution from arginine to cysteine at position 316 (R316C). In mice, the same arginine residue is located at position 342. To establish the causal relationship between the mutation and the skeletal phenotype observed in the patients, we generated a knock-in mouse model where the amino acid variant (R316C) was introduced to position 342 in the mouse *Sp7* gene (Fig. 1a) using the iGONAD method.^21,22^ A founder male was identified and then mated with a female FvB mouse to establish the F1 progeny. Heterozygous F3 mice were intercrossed to generate homozygotes with corresponding littermate controls. Both PCR and Next-Generation sequencing confirmed the generation of *Sp7*^*R342C*^ knock-in mice (Fig. 1b, c).

### *Sp7*^*R342C*^ mice exhibit lower bone mineral density and higher cortical porosity compared to wild-type littermates

**Fig. 2:**
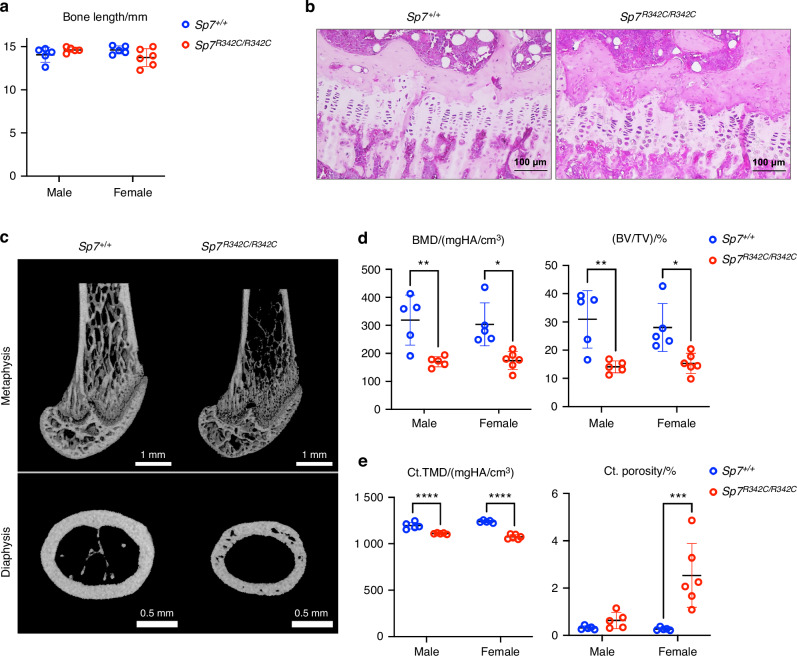
Bone loss and cortical porosity in *Sp7*^*R342C*^ knock-in mice. **a** *R342C* mutation does not affect femur length of 8-week-old mice. **b** H&E-stained paraffin-embedded sections from the tibia show no growth plate morphological differences between *Sp7*^*R342C/R342C*^ mice and wild-type littermates. Scale bars are shown in the images. **c** μCT images from the femoral metaphysis and diaphysis of 8-week-old mice show bone loss and increased cortical porosity in *Sp7*^*R342C/R342C*^ mice compared to wild-type littermates. Scale bars are shown in the images. **d** Quantification of metaphysis parameters. **e** Quantification of midshaft diaphysis. BMD: bone mineral density, BV/TV: bone volume fraction, Ct. TMD: cortical bone tissue mineral density, Ct. porosity: cortical porosity. Two-way ANOVA analysis followed by post hoc Tukey–Kramer test was performed (*P < 0.05; **P < 0.01, ***P < 0.001, ****P < 0.000 1)

*Sp7*^*R342C*^ mice are viable, born at expected Mendelian ratios, and show normal skeletal patterning, growth, bone length, and growth plate morphology (Fig. 2a, b, Table S2). To examine the skeletal phenotype of *Sp7*^*R342C*^ mice, we first performed micro-CT imaging in the femur of 8-week-old mice. Compared to littermate controls, male and female mutant *Sp7*^*R342C/R342C*^ mice demonstrated lower bone mineral density and trabecular bone volume fraction in the metaphysis, higher cortical porosity, and reduced diaphyseal cortical tissue mineral density (Fig. 2c–e, Table S2). These findings are consistent with phenotypes observed in patients homozygous for this mutation.^16^

To test whether the *R342C* mutation affects bone strength, three-point bending was performed on femurs from female mice of each genotype to measure the whole bone and apparent tissue-level mechanical properties. We performed the test in female mice due to more dramatic bone phenotypes observed in female *Sp7*^*R342C/R342C*^ mice (Fig. 2e, Table S2). The femurs of *Sp7*^*R342C/R342C*^ mice show no statistically significant changes in bending rigidity and apparent bending modulus than the femurs of wild-type littermates and reach similar ultimate moments and apparent ultimate stresses as the wild-type femurs (Fig. S1a, Table S3). However, mutant femurs showed increased post-yield properties with significantly greater post-yield displacement (+109%, *P* = 0.048) and post-yield work to fracture (+92%, *P* = 0.023) than the femurs of WT mice (Fig. S1b). The femurs of *Sp7*^*R342C/R342C*^ mice exhibited greater energy absorption capacity than the wild-type femurs by requiring greater work to yield (+54%, *P* = 0.036) and fracture (+82%, *P* = 0.015) (Fig. S1b). These results suggest that the disease associated *Sp7* mutation, which reduces cortical tissue mineral density, may cause bone to become more ductile with greater energy absorption capacity than samples from WT mice.

### Osteocyte dendrite defects in *Sp7*^*R342C*^ mice

**Fig. 3:**
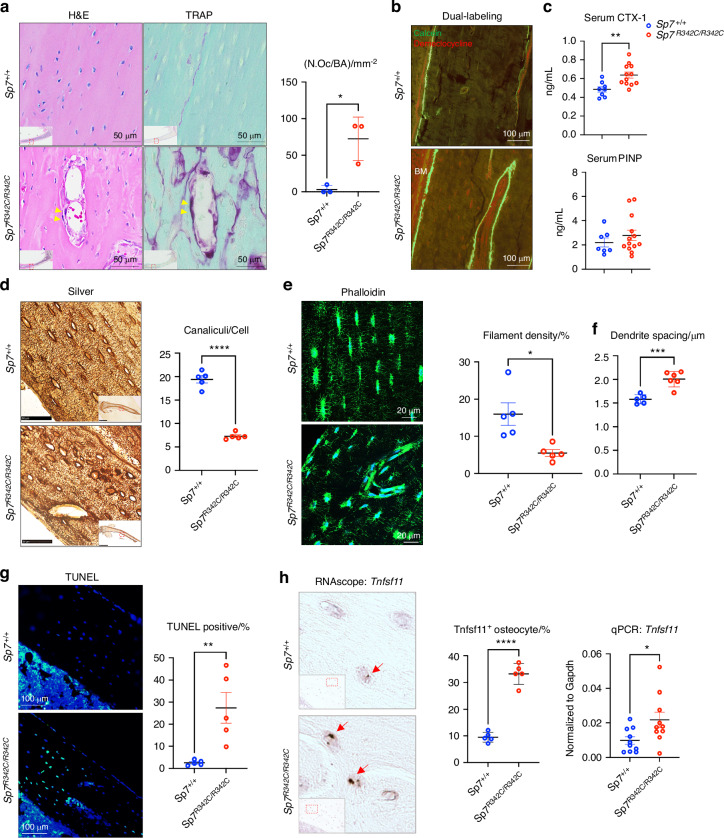
*Sp7*^*R342C*^ mice show osteocyte dendrite defects and increased osteoclast activity. **a** TRAP-stained (red) paraffin-embedded sections from the tibia show an increased number of TRAP^+^ multinucleated intracortical osteoclasts in *Sp7*^*R342C*^ in mice (yellow arrowheads). H&E staining was done in the serial paraffin-embedded sections. Bottom left: low magnification image showing the staining of the whole section. The region with red dashed lines is zoomed in. Right: Quantification of TRAP^+^ multinucleated osteoclasts per bone area (N.Oc/BA) in the cortical bone with Image J. **b** 8-week-old mice were labeled with calcein (green) and demeclocycline (red) 7 and 2 days prior to sacrifice, respectively. Non-decalcified sections from the cortical bone in the femur were analyzed. Control mice show normal endosteal bone formation. In contrast, *Sp7*^*R342C*^ animals show abnormal intracortical bone formation. **c** Serum CTX-1 level is significantly increased in *Sp7*^*R342C/R342C*^ female mice. There is a trend of increasing serum P1NP level in *Sp7*^*R342C/R342C*^ female mice. **d** Silver nitrate staining shows reduced canaliculi number per cell in mutant mice versus controls, quantified on the right. Bottom right: low magnification image showing the staining of the whole section. The region with red dashed lines is zoomed in. **e** Cryosections from 8-week-old control and *Sp7*^*R342C*^ tibia were stained with phalloidin to visualize actin filaments. Quantification (right) indicates reduced filament density (percent of acellular bone matrix occupied by phalloidin-positive filaments). **f** Increased dendrite spacing was observed in *Sp7*^*R342C*^ mice. **g** Apoptosis in situ was analyzed on tibia sections by TUNEL staining and demonstrates increased osteocyte apoptosis in *Sp7*^*R342C*^ mice versus controls. Green: TUNEL; Blue: DAPI. **h** In situ hybridization (RNAscope) shows increased *Tnfsf11* expression in *Sp7*^*R342C/R342C*^ osteocytes (red arrows). Bottom left: low magnification image showing the staining of the large section. The region with red dashed lines is zoomed in. *Tnfsf11*^*+*^ osteocytes are pointed by red arrows. There is a significant increase of *Tnfsf11*-positive osteocytes in mutant mice based on RNAscope staining. Right: RT-qPCR performed in the enriched osteoblasts and osteocytes isolated from humerus (without bone marrow) confirms the increased expression of *Tnfsf11* in *Sp7*^*R342C*^ mice. Student’s *t* test was performed for statistical analysis (*P < 0.05; **P < 0.01, ***P < 0.001, ****P < 0.000 1). Scale bars are shown in the images

*Sp7*^*R342C*^ knock-in mice showed abnormal intracortical bone remodeling as characterized by increased intracortical bone resorption and formation (Fig. 3a, b) associated with increased serum levels of the bone resorption marker CTX-1 without significant changes in the bone formation marker P1NP (Fig. 3c). Further supporting the notion that increased intracortical osteoclastic remodeling is present in mutant mice, cortical bone RNA shows increased expression of several osteoclast markers (Table S4: *Acp5, Ctsk, Calcr, Nfatc1*). Silver nitrate staining of the lacunar-canalicular network (LCN) showed that bones from *Sp7*^*R342C*^ mutant mice have reduced canalicular number per osteocyte (Fig. 3d). In situ phalloidin staining of actin filaments demonstrated reduced osteocyte dendrites in *R342C* mutant mice (Fig. 3e). To determine if this reduction in canalicular number, observed in 2D, resulted from canalicular loss or changes in canalicular tortuosity, we examined osteocyte dendrites in 3D using thick sections of phalloidin-stained cleared cortical bone. *R342C* mutant mice possess fewer dendrites per cell, with increased dendrite spacing (Fig. 3f). The loss of dendrites is sufficiently profound to cause a reduction of filament density, despite increased osteocyte density and reduced dendrite tortuosity in *Sp7*^*R342C*^ mutant mice compared to controls (Fig. 3d–f, Fig. S2a). Osteocyte apoptosis was elevated in mutant mice as assessed by terminal deoxynucleotidyl transferase dUTP nick end labeling (TUNEL) staining (Fig. 3g). Increased percentage of *Tnfsf11*^+^ (the gene encoding Rankl, the key osteoclastogenic cytokine) osteocytes was observed in osteocytes near intracortical bone remodeling region by in situ hybridization, and RT-qPCR performed in enriched osteoblasts and osteocytes isolated from the humerus cortical bone confirmed increased *Tnfsf11*^+^ expression in mutant mice (Fig. 3h). Therefore, the *Sp7 R342C* mutation results in osteocyte dendrite defects and osteocyte apoptosis.

### *R342C* mutation does not affect osteoblast differentiation in vitro

**Fig. 4:**
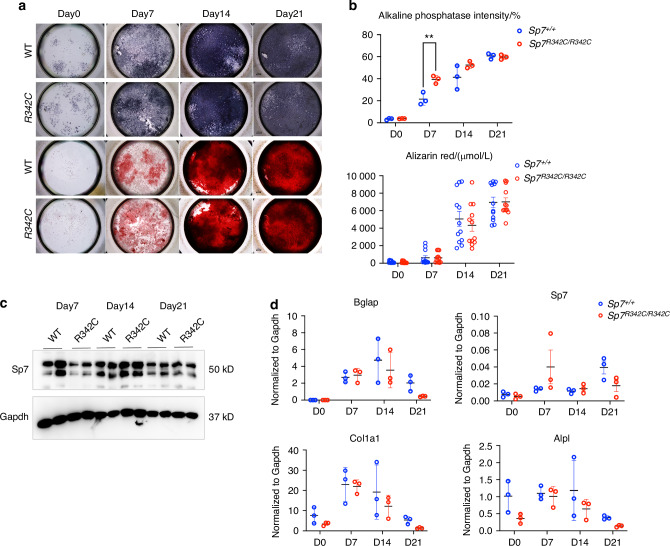
*Sp7*^*R342C*^ mutation does not affect BMSC differentiation into osteoblasts. **a** Bone marrow stromal cell (BMSC) differentiation was examined with alkaline phosphatase staining (top) and alizarin red staining (bottom). BMSCs were seeded at the same density (50 000 cells/mL) and the osteogenic differentiation were induced by L-ascorbic acid and β-glycerophosphate. **b** Quantification of alkaline phosphatase and alizarin red staining show no differences between mutants (*Sp7*^*R342C/R342C*^) and controls (*Sp7*^*+/+*^). Each dot represents one biological replicate. **c** Western blot of BMSCs show that the protein level of Sp7 is not affected by the *R342C* mutation. Two biological replicates were performed per genotype per time point. **d** RT-qPCR was performed in differentiated BMSCs. Several osteoblast markers were tested and there is no expression difference between wild-type and *R342C* mutant BMCSs. Student’s *t* test was performed for statistical analysis between WT and mutant cells at each time point (*P < 0.05; **P < 0.01, ***P < 0.001, ****P < 0.000 1). Scale bars are shown in the images. All images in the same panel have the same scale

To examine whether the *R342C* mutation affects the osteoblast differentiation function of Sp7, we isolated bone marrow stromal cells (BMSCs) from 8-week-old mice of both genotypes and cultured them in osteogenic media (L-ascorbic acid and β-glycerophosphate) in vitro. After 4 weeks of differentiation, both alkaline phosphatase staining and alizarin red staining showed no significant difference between cells obtained from *Sp7*^*R342C/R342C*^ mice and control littermates (Fig. 4a). Quantification of alkaline phosphatase staining and alizarin red staining, markers of osteoblastic matrix mineralization, confirmed similar levels between mutant mice and control littermates (Fig. 4b). Western blot demonstrated that Sp7 protein levels do not differ in BMSCs from *Sp7*^*R342C/R342C*^ mice and wild-type controls (Fig. 4c). Furthermore, comparable patterns of osteoblast marker gene expression (*Bglap, Sp7, Col1a1, Alpl*) were noted between BMSCs in *Sp7*^*R342C/R342C*^ mice and wild-type controls (Fig. 4d). Thus, although osteocytes bearing the *Sp7*^*R342C*^ mutation show morphology and survival defects in vivo, this mutation does not grossly impair osteoblast function in vitro.

### Osteocyte genes are dysregulated in *Sp7*^*R342C*^ mice

**Fig. 5:**
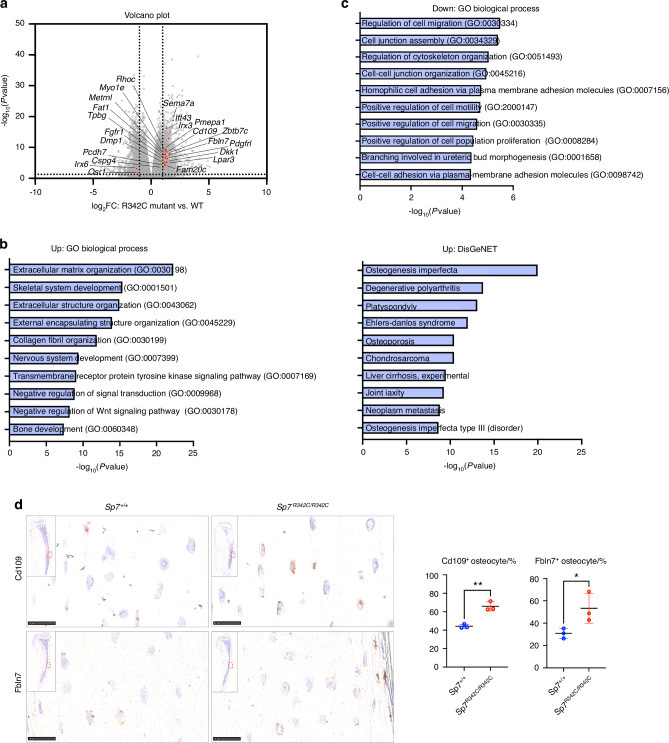
Globally-dysregulated osteocyte gene expression in *R342C* mice. **a** Volcano Plot of differentially expressed genes. 22 overlapping osteocyte signature genes are highlighted in red. **b**–**c** Gene Ontology of genes that were up-regulated (**b**) and down-regulated (**c**) in *R342C* mutant mice. Differentially expressed genes were identified with DEseq2 (*P*-adj < 0.05 and |log_2_FC | ≥ 1). 1 079 genes were up-regulated and 920 genes were down-regulated. DisGeNET: the database of gene-disease associations. **d** In situ hybridization of *Cd109* and *Fbln7* in tibial paraffin sections of 10-week-old mice. Top left: low magnification image showing the staining of the whole section. The region with red dashed lines is zoomed in. Right: *Cd109-* and *Fbln7-*positive osteocytes were quantified. Scale bar: 25 μm

To identify the genes that are dysregulated by the *R342C* mutation, we performed bulk RNA-seq analysis in marrow-flushed cortical bone RNA isolated from the humeri of female *Sp7*^*R342C/R342C*^ mice and control littermates. 1 079 genes were up-regulated and 920 genes were down-regulated in *Sp7*^*R342C/R342C*^ mice (*P*-adj < 0.05 and |log_2_FC | ≥ 1) (Fig. 5a). Gene Ontology (GO) revealed that up-regulated genes are linked to “Extracellular Matrix Organization”, “Skeletal System Development” in GO Biological Process and “Osteogenesis Imperfecta” and “Osteoporosis” in DisGeNET (Fig. 5b). Down-regulated genes are significantly enriched in terms like “Regulation of Cell Migration” and “Regulation of Cytoskeleton Organization” (Fig. 5c). To extract the osteocyte genes that are affected by this mutation, we intersected these dysregulated genes identified from bulk RNA-seq with the top 500 osteocyte markers identified from our previous single-cell RNA-seq analysis,^13^ and the ‘osteocyte transcriptome’ gene set reported by Youlten et al.^23^ We identified 22 overlapping osteocyte genes (Fig. 5a, Table S4). Among the shared genes, 20 genes were up-regulated and 2 genes were down-regulated in the *R342C* mutant. Several genes in this osteocyte marker set are up-regulated in mutant bone RNA, including *Fbln7*, *Cd109*, *Dmp1*, *Irx3*, *Irx6* and *Dkk1* (Fig. 5a). We previously identified *Fbln7* as a top marker gene from the *Sost*-positive osteocyte subpopulation. *Fbln7* is expressed in both newly formed osteocytes and mature osteocytes,^13^ and in situ hybridization confirms the increased expression level of *Fbln7* in mutant osteocytes (Fig. 5d). *Cd109*, a GPI-anchored cell-surface glycoprotein involved in TGFß signaling identified as a causal gene for osteoporosis,^24,25^ also shows increased expression in mutant osteocytes (Fig. 5d).

To evaluate osteoblast activity, we performed static and dynamic histomorphometry in the femur of 8-week-old female mice. *Sp7*^*R342C/R342C*^ mice had increased osteoblast numbers, increased osteoid content, and increased mineralization lag time (Fig. S2b, c, Table S5), findings that are consistent with phenotypes observed in *R316C* patients.^16^ Although small amounts of unmineralized matrix is present in control animals, osteoid amounts are reported as absent in control mice due to technical sensitivity of this measurement. Mineral apposition rate and bone formation rate were significantly reduced in *Sp7*^*R342C/R342C*^ mice (Fig. S2c). Serum PTH, phosphate and calcium levels are not affected in *Sp7*^*R342C/R342C*^ mice compared to controls (Fig. S2d). These histomorphometry findings suggest that the *R342C* mutation may interfere with the function of mature osteoblasts and embedding osteocytes to promote matrix mineralization.

### Osteocyte dendrite defects caused in *Sp7*^*R342C*^ mice are independent of osteoclast activity

**Fig. 6:**
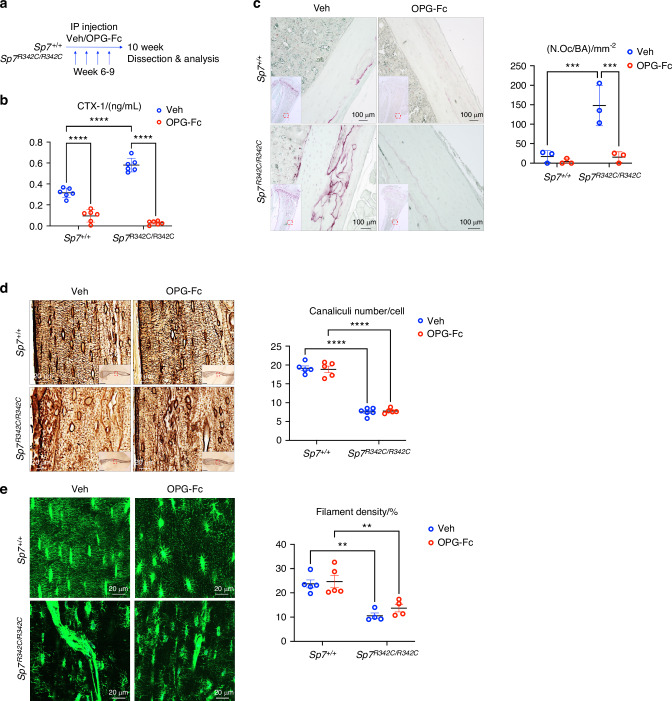
Osteocyte dendrite defects caused by *R342C* mutation are independent of osteoclast activity. **a** Schematic illustration of OPG-Fc injection. **b** Serum CTX-1 level is significantly reduced in OPG-Fc injected wild-type and *R342C* mutant mice. **c** TRAP staining of decalcified, paraffin-embedded tibia sections reveals decreased osteoclasts in both wild-type and *Sp7*^*R342C*^ mice following OPG-Fc treatment. Bottom left: low magnification image showing the staining of the whole section. The region with red dashed lines is zoomed in. Right: Quantification of TRAP^+^ multinucleated osteoclasts per bone area (N.Oc/BA) in the cortical bone with Image J. **d** Silver nitrate staining shows no difference in the canaliculi number per cell between OPG-Fc and vehicle-treated *Sp7*^*R342C*^ mice. Bottom right: low magnification image showing the staining of the whole section. The region with red dashed lines is zoomed in. **e** Phalloidin staining shows no difference in the dendrite filament density between OPG-Fc and vehicle-treated *Sp7*^*R342C*^ mice. Two-way ANOVA analysis followed by post hoc Tukey–Kramer test was performed (*P < 0.05; **P < 0.01, ***P < 0.001, ****P < 0.000 1). Scale bars are shown in the images

In multiple instances, osteocyte morphology defects and osteocyte apoptosis are accompanied by increased intracortical bone resorption by osteoclasts.^13,26,27^ However, the causal relationship between these two findings remains unclear. Specifically, it is not known whether osteocyte morphology defects and apoptosis drive bone resorption, or whether increased osteoclast activity drives osteocyte morphology defects. *Sp7 R342C* mutant mice provides an ideal model to test this important question in bone cell communication. Therefore, we assessed if the dendrite defects seen in *Sp7*^*R342C*^ mice are dependent on osteoclast activity by treating mice with OPG-Fc, a potent inhibitor of RANKL-driven bone resorption.^28–30^ Female mutant *Sp7*^*R342C/R342C*^ mice and their control littermates were injected once weekly with OPG-Fc^31^ or saline starting from 6 to 9 weeks of age (Fig. 6a). A three-week treatment period was selected because this time frame was sufficient to observe improvements in osteocyte morphology upon osteocrin administration in our previous work with *Sp7* conditional knockout mice.^13^ OPG-Fc treatment showed expected effects including reduced serum CTX-1 and increased trabecular bone mass in both wild-type and *Sp7*^*R342C/R342C*^ mice (Fig. 6b, Fig. S3, Table S6). While cortical porosity tended to be increased in mutant mice and reduced by OPG-Fc treatment, these differences were not statistically significant. TRAP staining revealed reduced intracortical osteoclasts in mice with OPG-Fc treatment compared to vehicle treatment (Fig. 6c). In contrast, osteocyte dendrite defects and disrupted lacunar-canalicular network (LCN) in *Sp7*^*R342C/R342C*^ mice were not rescued with OPG-Fc treatment (Fig. 6d, e). Thus, increased intracortical bone resorption is not a primary driver of osteocyte morphology defects in the *Sp7 R342C* mutant mice.

## Discussion

Here we report the generation of *Sp7*^*R342C*^ mice as a model for osteogenesis imperfecta due to *SP7 R316C* mutation in humans. These animals recapitulate many aspects of skeletal disease in humans with this mutation including increased cortical porosity, largely preserved markers of bone formation, and osteocyte dendrite defects. Traditionally, osteogenesis imperfecta (OI) has been viewed as a disease due to defects in collagen production and processing, functions typically ascribed to osteoblasts and their progenitors.^32,33^ Although some reports have noted changes in osteocyte density and osteocyte morphology in OI models,^32–34^ these defects in osteocyte parameters have occurred in the setting of dramatic changes in osteoblast function. Our model is intriguing in that early osteoblast lineage commitment is largely preserved, yet a skeletal phenotype exists with most apparent changes being driven by mature osteoblasts and osteocytes. Thus, these results provide a new framework for OI pathogenesis which focuses on lineage stage-specific roles of Sp7 in mature osteoblasts and osteocytes.

Further functional studies are needed to understand how the *SP7 R316C* mutation affects the function of this transcription factor. The observation that mature osteoblasts and osteocytes appear to be more sensitive to this mutation than early osteoblast progenitors raises the hypothesis that cell type-specific SP7 binding partners or post-translational modifications may exist. Other *SP7* point mutations (*1502delA*, *E340A*, and *S309W*) have been associated with distinct skeletal dysplasia in humans.^17,18,35,36^ Notably, the *S309W* human mutation, a change at a site adjacent to the *R316C* residue studied here, causes a distinct phenotype associated with dysregulated osteoblast maturation and a shifted DNA binding profile.^36^ Thus, defining the impact of the *R316C* mutation on DNA binding patterns across the genome represents an important priority.

Although serum P1NP levels, a marker of bone formation by osteoblasts, are comparable between control and *Sp7*^*R342C*^ homozygous mutant mice (Fig. 3c), we do note changes in mature osteoblast function in histomorphometry results (Table S5, Fig. S2b, c). Specifically, mutant mice show modest increases in osteoblast numbers with increased osteoid volume and reduced bone formation rate. Increased osteoidosis in *Sp7*^*R342C*^ mutants may lead to more ductility observed in biomechanical testing (Fig. S1). These findings suggest that mineralization defects during terminal osteoblast maturation and/or cells undergoing the osteoblast-to-osteocyte transition may be present. Defining the effects of this *Sp7* point mutation on gene expression programs in distinct subpopulations of mature osteoblasts and developing osteocytes remains an exciting investigative topic.

The precise Sp7 target genes responsible for defects in osteocyte morphology and survival reported here are not known. Bone transcriptomic analysis (Fig. 5a, Table S4) demonstrated dysregulated expression of many osteocyte marker genes in *Sp7*^*R342C*^ mutant mice versus controls. Surprisingly, this analysis showed increased levels of many osteocyte marker genes, findings confirmed by in situ hybridization. Currently, we interpret these findings as a compensatory attempt by osteocytes to restore normal maturation in light of morphology defects. However, we cannot rule out a model in which Sp7 normally inhibits expression of a network of genes expressed by terminally-differentiated osteocytes. Defining the impact of this *Sp7* point mutation on genome-wide binding in light of these transcriptomic changes is needed to gain insight into how Sp7 controls osteocyte maturation.

Additional limitations of the current study are noted. First, the *Sp7* point mutation is present in all cells using our knock-in strategy, thus rendering it difficult to precisely decipher the contribution of mature osteoblasts versus osteocytes towards the skeletal phenotype that is observed. Future studies using conditional ‘knock-in’ models will be necessary to address this question. Second, the relative roles of osteocyte morphology defects, dysfunction of osteoblastic matrix mineralization, and indirect increases in intracortical osteoclastic bone resorption to the overall skeletal phenotype observed in *Sp7*^*R342C*^ knock-in mice remain to be determined. It is likely that, in osteocytes, morphology defects trigger *Tnfsf11* upregulation and apoptosis which promote intracortical remodeling and cortical porosity. In trabecular bone, defects in osteoblastic matrix mineralization may underlie the cancellous osteopenia that is observed. As noted above, future studies involving a conditional point mutant model will be needed to define the relative contributions of closely-related *Sp7*-expressing cells towards these different skeletal phenotypes. Third, we acknowledge that the study design with OPG-Fc leaves open the possibility that osteoclasts may influence the morphology of newly-formed osteocytes rather than pre-existing, matrix-embedded cells. Future studies in which OPG-Fc treatment is initiated earlier in life will be performed to address this open question. Fourth, analysis of the phenotype of *Sp7*^*R342C*^ mice was performed at a single time point (~8 weeks old) in hindlimbs. This mouse age was selected because patients with this mutation present with skeletal fragility early in life. Assessing the impact of this mutation on bone mass and bone quality as animals age and at different skeletal sites will be an important focus for future study. Finally, effects of *SP7* mutation at the level of osteocrin expression differ when the mutation is studied using over-expression versus knock-in approaches. In our previously reported overexpression-based studies,^13^ *SP7 R316C* was less effective at inducing *Ostn* enhancer activity and endogenous Ostn expression upon heterologous introduction. Here, although Ostn expression is clearly reduced by *Sp7* deletion in vivo,^13^ we do not observe obvious changes in Ostn levels in between control and knock-in mutant mice. Thus, it is critical to test overexpression-based findings using endogenous gene manipulations, as we have done here.

Development of this novel mouse model of osteogenesis imperfecta (OI) affords new opportunities to study mechanisms of disease and potential therapies. Though OI represents the classical example of bone with dysregulated material properties, and this aspect of bone quality is osteocyte-dependent, the role of osteocytes in OI-related bone fragility remains unclear. Additional study will be needed to uncover biological and material mechanisms by which the changes in *Sp7*^*R342C*^ osteocyte function deregulate the control of bone quality. While OPG-Fc treatment (Fig. 6) successfully reduced bone resorption markers, this agent did not rescue osteocyte morphology defects. Testing the efficacy of other agents which target cells in the osteoblast/osteocyte lineage such as parathyroid hormone,^37^ anti-sclerostin antibody,^38^ anti-TGFβ antibody,^39^ or osteocrin^13^ remains an important priority for future studies. Furthermore, our findings here highlight the need to assess osteocyte morphology and function defects in other forms of osteogenesis imperfecta.

## Materials and methods

### Mice

*R342C* knock-in mice were generated by the CRISPR-mediated in vivo genome editing, i-GONAD,^21^ using the FvB outbred strain (Charles River Laboratory). Briefly,^21,22^ at 0.7 day post coitum, the female was anesthetized by continuous inhalation of isoflurane. Through a 1 cm long skin incision, a 0.5 cm incision of each side was made to the muscle layer to access the abdominal cavity. The fat pad attached to the ovary was identified and pulled outside of the body to expose the oviduct. 1.5 μL of CRISPR solution containing 1 μg/μL of Cas9 (PNA Bio) premixed with 10 mmol/L two-piece guide RNA (crRNA and tracrRNA) (IDT) with or without 1 μg/μL of a single strand DNA repair template, was injected into the oviduct either by puncturing or through the infundibulum. Then, in vivo electroporation was performed using the square pulse generator, BTX-820 with the electrode tweezers, CUY652P2.5×4 (Nepa Gene, Japan) with the setting of 50 V, 5 msec each pulse, 8 pulses, with a 0.5 cm electrode gap. After suturing the muscle layer and skin, the pregnant mother was returned to the cage. Pups were genotyped for desired modifications, then mated with FvB wild type mice to establish the F1 lines. Heterozygous F3 mice were intercrossed to generate homozygotes. The WT and repair template sequences are shown in Fig. 1a. At position 342, the codon for the mutant repair template was changed to TGT, which encodes cysteine. The remaining silent mutations in the repair template were introduced to limit further genome editing once initial recombination occurred. We also designed a ‘wild type’ repair template with only silent mutations to prevent deleterious editing of the non-targeted allele; however, germline transmission of this sequence was not observed. crRNA sequence is listed in Table S1. Genotypes were determined by PCR using primers listed in Table S1. All the experimental mice generated and used were after F3 crosses. Littermate controls were used for studies. Since humans with *SP7 R316C* mutations present with skeletal disease when this mutation is present in the homozygous state, analysis of mice here focused on comparisons between wild type and homozygous mutant mice. Both males and females were included in this study. All procedures involving animals were performed in accordance with guidelines issued by the Institutional Animal Care and Use Committees (IACUC) in the Animal Resource Center at University of Texas Southwestern Medical Center and the Center for Comparative Medicine at Massachusetts General Hospital under approved Animal Use Protocols (2024103714, 2019N000201). All animals were housed in the Animal Resource Center at University of Texas Southwestern Medical Center and the Center for Comparative Medicine at Massachusetts General Hospital (21.9 °C ± 0.8 °C, 45% ± 15% humidity, and 12-h light cycle 7 am–7 pm). For OPG-Fc treatment, 6-week-old *Sp7*^*R342C/R342C*^ and *Sp7*^*+/+*^ female mice were injected with either PBS or OPG-Fc (1 mg/mouse) once per week for 4 weeks by intraperitoneal injection.^31^ Mice were dissected at 10 weeks old for tissue collection.

### Micro-computed tomography (µCT)

Femurs were harvested from 8-week-old and 10-week-old mice after being fixed with 10% neutral buffered formalin for 1 day and stored in 70% ethanol. µCT imaging was performed on a bench-top scanner (µCT 40, Scanco Medical AG, Brüttisellen, Switzerland) to measure the trabecular and cortical bone architecture and mineral density of the femoral distal metaphysis and mid-diaphysis, respectively. Scans were acquired with 10 μm isotropic voxels, 70 kVp peak x-ray tube potential, 114 mA intensity, and 200 ms integration time. Trabecular bone was analyzed in the endocortical region of the distal metaphysis beginning 200 µm superior to the distal growth plate and extending 1.5 mm (150 transverse slices) proximally. The bone within the trabecular region of interest was segmented using a threshold of 390 mgHA/cm^3^ and analyzed using the Scanco IPLV6 Trabecular Bone analysis script. Trabecular architecture outcomes included trabecular bone volume fraction (Tb.BV/TV,%), trabecular bone mineral density (Tb.BMD, mgHA/cm^3^), trabecular thickness (Tb.Th, mm), trabecular number (Tb.N, mm^-1^), trabecular separation (Tb.Sp, mm), connectivity density (ConnD, mm^-3^), Cortical architecture was analyzed in a 500 μm long region (50 transverse slices) of the mid-diaphysis. The bone within the cortical region of interest was segmented using a threshold of 700 mgHA/cm^3^ and the geometry of the cortex analyzed using the Scanco Midshaft Evaluation script. Cortical architecture outcomes included total area (Tt.Ar, mm^2^), cortical bone area (Ct.Ar, mm^2^), medullary area (Ma.Ar, mm^2^), cortical bone area fraction (Ct.Ar/Tt.Ar, %), cortical tissue mineral density (Ct.TMD, mgHA/cm^3^), cortical thickness (Ct.Th, mm), cortical porosity (%), as well as the maximum, minimum and polar moments of inertia (I_max_, I_min_, and pMOI, mm^4^), which describe the shape and distribution of cortical bone (larger moments of inertias generally indicate greater rigidity). Cortical porosity was measured as the total area of pores in the cortex (pore area = cortex area – Ct.Ar) divided by the total area of the cortex [cortical porosity/% = (pore area/cortex area) × 100)].

### Three-point bending test

Femurs from 10-week-old female mice were collected and were mechanically tested in three-point bending using an electrical-force material testing machine (Electroforce 3230, Bose Corporation, Eden Prairie, MN). Femurs were wrapped in saline soaked gauze and store at −20 °C from the time of harvest to testing. Prior to testing, µCT analysis was performed to measure the mid-diaphysis geometry of the femurs using the same methods described in the previous µCT section. The bending fixture had a bottom span length of 8 mm. The test was performed with the load point in displacement control moving at a rate of 0.1 mm/s with force and displacement data collected at 60 Hz. All the bones were positioned in the same orientation during testing with the cranial surface resting on the supports and being loaded in tension. Bending rigidity (EI, N-mm^2^), apparent modulus of elasticity (E_app_, MPa), ultimate moment (M_ult_, N-mm), apparent ultimate stress (σ_app_, MPa), work to yield and fracture (W, mJ), post-yield displacement (mm), and apparent toughness (U_app_, mJ/mm^3^) were calculated based on the force and displacement data from the tests and the mid-shaft geometry measured with µCT. The minimum moment of inertia (I_min_) and bone radius perpendicular to I_min_ (C_min_) were measured with µCT and were used when calculating the apparent material properties. Work to yield and fracture are the energies required to cause the femur to yield and fracture, respectively, and were calculated by finding the area under the force-displacement curve using the Riemann Sum method. Yield was determined by finding the intersection of the load-displacement curve and a regression line with a 10% lower slope than the linear portion of the force-displacement curve.^40^ Bending rigidity was calculated using the linear portion of the force-displacement curve.

### Histology and histomorphometry

Formalin-fixed paraffin-embedded decalcified tibia sections from 8-week-old and 10-week-old mice were obtained. Hematoxylin and eosin staining were performed using standard protocols. Briefly, sections were deparaffinized and rehydrated. Sections were then stained with Tacha’s CAT Hematoxylin (Biocare Medical) for 1 min followed by washing. Finally, sections were counterstained in Alcoholic-Eosin (1% eosin y, 1% phloxine B, 95% ethanol, glacial acetic acid) for 1 min and followed by dehydration.

Right femurs from 8-week-old female mice have been subjected to bone histomorphometric analysis (calcein and demeclocycline labeling). All mice received intraperitoneal calcein (Sigma-Aldrich, 20 mg/kg) and demeclocycline (Sigma-Aldrich, 40 mg/kg) injections at 7 days and 2 days prior to sacrifice. The tibia was dissected and fixed in 70% ethanol for 3 days. Fixed bones were dehydrated in graded ethanol, then infiltrated and embedded in methyl methacrylate without demineralization. Undecalcified 5 μm longitudinal sections were obtained using a microtome (Leica Biosystems, RM2255). Histomorphometry analyses were performed according to the criteria established by the American Society of Bone and Mineral Research.^41^ Trabecular static, structural, and dynamic parameters were measured in the distal femoral metaphysis, 0.2 mm below the epiphyseal growth plate, using an Osteomeasure image analyzer (Osteometrics). All analyses were performed in a blinded fashion. Digital images were obtained via fluorescent microscopy. Standard Masson’s trichrome staining was performed to discriminate between calcified and uncalcified bone and cartilage in sections.

### Silver staining and phalloidin staining

As described previously,^13,42^ paraffin-embedded mouse tibia sections were deparaffinized and incubated in two parts, 50% silver nitrate and one part 1% formic acid in 2% gelatin solution for 55 min. Stained slides were then washed in 5% sodium thiosulfate for 10 min and subsequently dehydrated, cleared, and mounted. Stained slides were first scanned with Nanozoomer to capture the entire stained section. 4 images were taken at 40x within the medial tibia cortex. Quantification of the canaliculi number per cell were performed with ImageJ by thresholding grayscale images for dark, silver-stained lacunae and canaliculi.

Tibiae were submerged in 30% sucrose, and embedded in O.C.T. Compound (Fisher HealthCare), and cryo-sectioned. For 10 μm tibia cryosections, slides were first washed in cold PBS for 5 min, fixed with 4% PFA (Thermo Scientific) in PBS for 10 min and permeabilized with 0.05% Saponin (Thermo Scientific) in PBS for 5 min. Sections were then incubated with phalloidin mixture (1:1 000, Abcam) in the dark for 1 h at room temperature. Sections were washed with cold PBS three times before being stained with DAPI (1:1 000, Invitrogen) for 15 min, mounted with Fluoromount-G (Invitrogen) and imaged by confocal microscopy. 3 images per sample were taken within the medial cortex of the tibia. Quantification of osteocyte filament density was performed in ImageJ imaging software on a blinded basis. First, cell bodies were cropped, and then multi-color images were converted to single-channel (grayscale) color images. Each image was duplicated, and a binary image was created from the copied image. In the copied image, all dendrites were highlighted, and the background was subtracted. Next, we used “Analyze – Set Measurements” and set “Redirect to” to the original grayscale image, followed by selecting the “Area” and “Mean Gray Value” functions. Finally, the “Analyze – Analyze Particles” function was used to quantify the filament density.

### Fluorescent imaging of the lacunae canalicular network in 3D

Femurs (*n* = 5-6 per group) clean free of the periosteum and muscle was fixed with 10% neutral buffered formalin (NBF), decalcified in EDTA, and processed for cryosectioning as previously described.^42^ Briefly, decalcified bones underwent a series of sucrose gradient solutions (15%, 20%, 30%) followed by O.C.T. embedding. A cryostat was used to obtain 50 µm thick axial sections of the mid-cortical femurs and stained for the osteocyte cell membrane with hydrophobic lipophilic membrane dye 1,1’-dioctadecyl-3,3,3’,3’-tetramethylindocarboxyanine percholate (DiI) (Invitrogen), actin cytoskeleton with Alexa Fluor 488 phalloidin (Invitrogen), and nuclei with DAPI as previously described.^27,42–44^ Briefly, cryosections in a 48 well plate were rinsed in PBS to remove the O.C.T. media and blocked with blocking buffer (1% normal donkey serum in 1% bovine serum albumin in PBS) overnight, incubated in 100 μmol/L of DiI in 50% DMSO/50% PBS for 1 week, followed by 165 nmol/L phalloidin in blocking buffer incubation overnight, and lastly with DAPI for 30 min at room temperature. Stained sections were cleared with 2,2’-thiodiethanol (TDE) gradient solutions (10%, 25%, 50%, 95%), mounted with 97% TDE under a number 1 coverslip and edges sealed with toluene/formaldehyde free nail polish. Stained sections (3 sections per mouse) were imaged using a Lecia DMi8 (Leica Microsystems) inverted confocal microscope utilizing LAS X software.

Quantification of dendrite tortuosity, dendrite spacing, and the lacunar-canalicular network (LCN) volume fraction in 3D was previously described^45^ using Fiji/ImageJ^46^ software plugins: BoneJ^47^ and Simple Neurite Tracer on 3D reconstruction of Z-stack 100x images stacks with 115–120 slices of the anterior/posterior region of the cortical femur transverse section. For dendrite tortuosity, the Simple Neurite Tracer generated the shortest pathway between two osteocytes as a path length. For dendrite spacing and LCN volume fraction, images were converted to 8-bit binary prior to quantification. BoneJ plugin was used to obtain ‘Thickness’ outcome for canalicular spacing and ‘Area/Volume Fraction’ outcome for the LCN volume fraction quantification. For quantification of osteocyte number, 40x images of the anterior/posterior region of the cortical femur transverse section were used to manually count the osteocytes using the Cell Counter plugin. The bone area was obtained and used to calculate the number of osteocytes per bone area for each sample.

Quantitative averages represent data collected from 3 technical replicates per mouse for 5–6 mice per group. Statistical comparison was calculated by unpaired two-tailed Student’s *t* test with Welch’s correction using Prism 10 (GraphPad Software Inc). Statistically significant is considered when *P*-value < 0.05 and values are shown as mean ± standard deviation.

### TUNEL staining

Formalin-fixed paraffin-embedded decalcified tibia sections from 8-week-old mice were obtained. For Terminal deoxynucleotidyl transferase dUTP nick end labeling (TUNEL), sections were fixed with 4% paraformaldehyde (PFA) (Pierce) in PBS for 15 min at room temperature and permeabilized with PCR grade recombinant Proteinase K (Roche Applied Science) for 30 min at 37 °C. Apoptotic cells were examined with Roche in situ cell death detection kit (Roche Applied Science) according to instructions and followed by 4′,6-diamidino-2-phenylindole (DAPI; Invitrogen) for fluorescent microscopy. 3 images per sample were taken within the medial cortex of the tibia.

### RNAscope

Formalin-fixed paraffin-embedded decalcified tibia sections (10 μm) from 8-week-old and 10-week-old mice were obtained. Bone sections were processed for RNA in situ detection using RNAscope 2.5 HD Assay-Brown (Chromogenic) according to the manufacturer’s instructions (Advanced Cell Diagnostics). For antigen retrieval, bone sections were pretreated with hydrogen peroxide and pepsin (1 h at 40 °C, Sigma-Aldrich). Tissue sections were hybridized with target probes (2 h at 40 °C), amplified (Amp1–4: 40 °C; Amp 5–6: room temperature) and chromogenic detected using DAB followed by counterstaining with hematoxylin (Millipore Sigma). RNAscope probes used were: *Tsfnf11* (NM_011613.3, REGION 314-1310), *Cd109* (NM_153098.3, region 632-1684) and *Fbln7* (NM_024237.4, region 198–1595). For the quantification of *Tnfsf11-*, *Cd109-* and *Fbln7*-positive osteocytes, 3 representative images were picked from the medial side of the cortical bone (3 mm below the growth plate) per mouse.

### TRAP staining

10 μm sections were first incubated in 0.2 mol/L acetate buffer (pH 5.0), then stained in the solution containing Naphthol AS-MX Phosphate (0.5 mg/mL; Sigma-Aldrich) and Fast Red TR Salt (1.1 mg/mL; Sigma-Aldrich) in 0.2 mol/L acetate buffer, and counterstained in Fast Green (Thermo Scientific Chemicals). The number of TRAP^+^ multinucleated osteoclasts within cortical bone was measured based on H&E and TRAP staining in serial sections. TRAP^+^ multinucleated cells were enumerated, and no acellular cement line or periosteal TRAP signals were quantified. Bone area was quantified with Image J. Measurement was set (Analyze > Set Measurements) and “Area” was selected. 3 images per sample were taken within the medial cortex of the tibia.

### Western blot

Whole-cell lysates were prepared using TNT (Tris-NaCl-Tween buffer, 20 mmol/L Tris-HCl pH = 8, 200 mmol/L NaCl, 0.5% Triton X-100 containing protease inhibitor (PI), 1 mmol/L NaF, 1 mmol/L DTT, 1 mmol/L vanadate). Adherent cells were washed with ice-cold phosphate-buffered saline (PBS), then scraped into TNT buffer on ice. The material was then transferred into Eppendorf tubes kept on ice, vortexed at top speed for 30 s, then centrifuged at top speed for 6 min at 4 °C. For immunoblotting, lysates were separated by sodium dodecyl sulphate–polyacrylamide gel electrophoresis, and proteins were transferred to nitrocellulose. Membranes were blocked with 5% dry milk blocker in tris-buffered saline plus 0.05% Tween-20 (TBST) and incubated with primary antibody overnight at 4 °C diluted in TBST plus 5% bovine serum albumin (BSA). The next day, membranes were washed, incubated with appropriate HRP-coupled secondary antibodies (Anti-rabbit IgG HRP-linked, Cell Signaling Technology, 1:2 000), and signals were detected with ECL Western Blotting Substrate (Pierce) or ECL Plus Western Blotting Substrate (Pierce). The primary antibodies were Sp7 (Abcam, dilution 1:1 000) and GAPDH (Cell Signaling Technology, dilution 1:1 000).

### ELISA

All serum measurement was performed using commercial bioassay detection reagents (IDS: P1NP, CTX-1, phosphate, PTH and calcium).

### RNA isolation and qRT-PCR

Right humerus was collected from 10-week-old mice. Muscle was carefully removed from the bone. Both ends of the humerus was cut to remove the trabecular bone. Bone marrow was flushed out and only the clean cortical bone was kept for RNA extraction. RNA was extracted by tissue blender with TRIzol (Invitrogen)/Chloroform based method, and further purification was performed with PureLink RNA mini column. cDNA was prepared with 1 µg RNA and synthesized using the PrimeScript RT Reagent Kit (TaKaRa). qPCR assays were performed on the StepOnePlus Real-time PCR System (Applied Biosystems) using PerfeCTa SYBR Green FastMix ROX (Quanta bio). *β*-actin was used as the internal control for normalization. The 2^−ΔΔCt^ method was used to detect expression fold change for each target gene with three biological replicates.

### RNA-seq analysis

Bone RNA samples were measured with High Sensitivity RNA ScreenTape analysis (Agilent). High quality RNA samples with RIN > 7 (*n* = 6) were processed for RNA library construction and sequencing using the Novoseq6000 platform (Novogene). Raw sequencing data were performed with quality control using FastQC/0.11.5. Transcripts were quantified using Salmon.^48^ Salmon index for mouse was generated based on the Ensembl fasta file for GRCm38. Differential expression analysis was performed using DESeq2^49^ package based on the criteria of false discovery rates (FDR) < 0.05 and log_2_|FC | ≥ 1. Analysis of gene ontology enrichment was performed using Enrichr on differentially expressed genes (https://amp.pharm.mssm.edu/Enrichr/)^50^ and the volcano plot was made using GraphPad Prism 9.

### BMSC differentiation

Culture and differentiation of bone marrow stromal cells (BMSCs) from mice were performed using standard protocols.^51^ Long bones were collected from 8-week-old mice. Briefly, small cuts were made at both the proximal and distal ends of the long bones (tibia and femur) before placing them in the sectioned tips within the centrifuge tubes. The marrow was collected at the bottom of the 1.5 mL tube by centrifuging at 10 000 × *g* for 15 s at room temperature. Cell pellets were suspended in 10 mL of BMSC culture media per mouse and filtered through the 70 μm filter. Cells were calculated and plated at 10^6^ cells/mL in 24-well plates (alizarin red staining) and 96-well plates (alkaline phosphatase staining, Sigma-Aldrich, SCR004). Cells were cultured at 37 °C until they reached 80%–100% confluency. BMSCs were differentiated by adding mineralization medium (50 μg/mL of L-ascorbic acid and 10 mmol/L beta-glycerophosphate). For staining and western blot, cells were analyzed after differentiated for 0, 7, 14 and 21 days.

### Alizarin red staining and alkaline phosphatase staining

For alizarin red staining, cells were fixed with ice-cold 70% ethanol for 1 h and stained with alizarin red stock solution (1% alizarin red in H_2_O, pH = 4.2) for 10 min (room temperature, rotation). For alizarin red staining quantification, cells were de-stain with 10% CPC for 15 min with rotation at room temperature. 200 μL of supernatant was transferred to 96-well plates and read at 562 nm. Alkaline phosphatase staining was performed following the manufactural protocol (Sigma-Aldrich). Alkaline phosphatase staining intensity was quantified with Image J. Images were first converted to 8-bit. Threshold was then set to the same for all images (Image > Adjust > Threshold > Apply). Measurement was set (Analyze > Set Measurements) and redirected to the 8-bit images. The relative intensity (%) was normalized to the total area and quantified (Analyze > Analyze Particles).

### Quantification and statistical analysis

All results are represented as mean ± SD for the indicated number of observations. Statistical details have been provided in the figures and figure legends. Differences between two groups were tested with two-tailed paired and unpaired Student’s *t* tests (GraphPad Prism 9). When more than two experimental groups were present, ANOVA analysis followed by post hoc Tukey–Kramer test was performed (GraphPad Prism 9). N represents mice or cell numbers as indicated for each experiment. A *P* < 0.05 was considered to be statistically significant.

## Supplementary information

Sup Figure 1

Sup Figure 2

Sup Figure 3

Table S1

Table S2

Table S3

Table S4

Table S5

Table S6

Supplementary legends

## Data Availability

The RNA-seq data is deposited in NCBI’s Gene Expression Omnibus (GEO) “GSE270443”. This paper does not report original code. The authors declare that all other data supporting the findings of this study are available within the article and its supplementary information files. Access to any additional information in this study is available upon request from the lead contacts, Jialiang S. Wang (jialiang.wang@utsouthwestern.edu) and Marc N. Wein (mnwein@mgh.harvard.edu).

## Material availability

Unique materials generated in this study are available upon request from the lead contacts, Jialiang S. Wang (jialiang.wang@utsouthwestern.edu) and Marc N. Wein (mnwein@mgh.harvard.edu).

## Code availability

The RNA-seq data is deposited in NCBI’s Gene Expression Omnibus (GEO) “GSE270443”. This paper does not report original code. The authors declare that all other data supporting the findings of this study are available within the article and its supplementary information files. Access to any additional information in this study is available upon request from the lead contacts, Jialiang S. Wang (jialiang.wang@utsouthwestern.edu) and Marc N. Wein (mnwein@mgh.harvard.edu).
