# Supplementary figures and images for: Osteoclast-independent osteocyte dendrite defects in mice bearing the osteogenesis imperfecta-causing Sp7 R342C mutation
Source: Bone Res. 2025 Jul 19;13:70. doi: 10.1038/s41413-025-00440-1 (PMC12276330; doi:10.1038/s41413-025-00440-1)

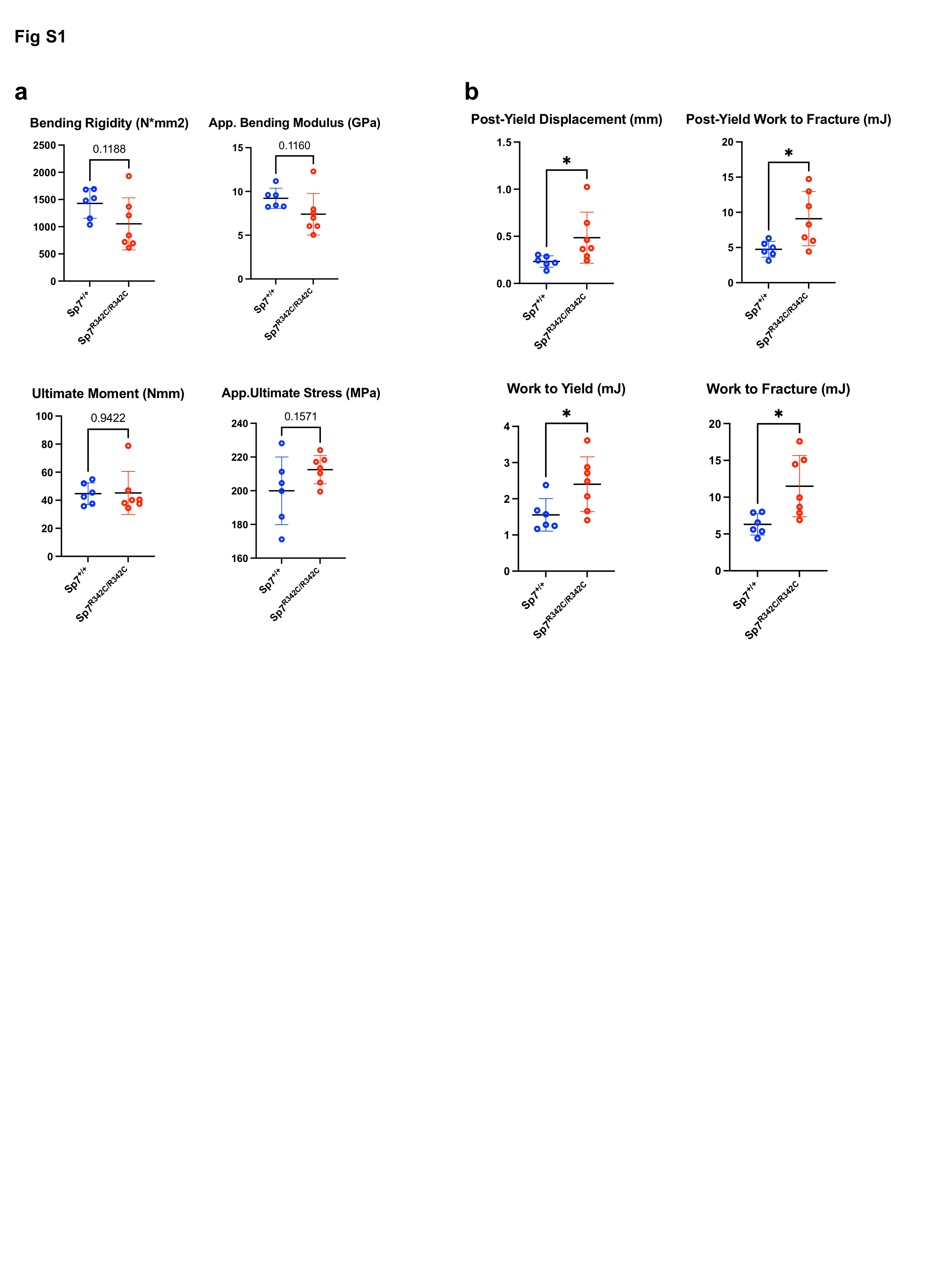

Supplement: Supplementary file 1 — Sup Figure 1 [file 41413_2025_440_MOESM1_ESM.tif]

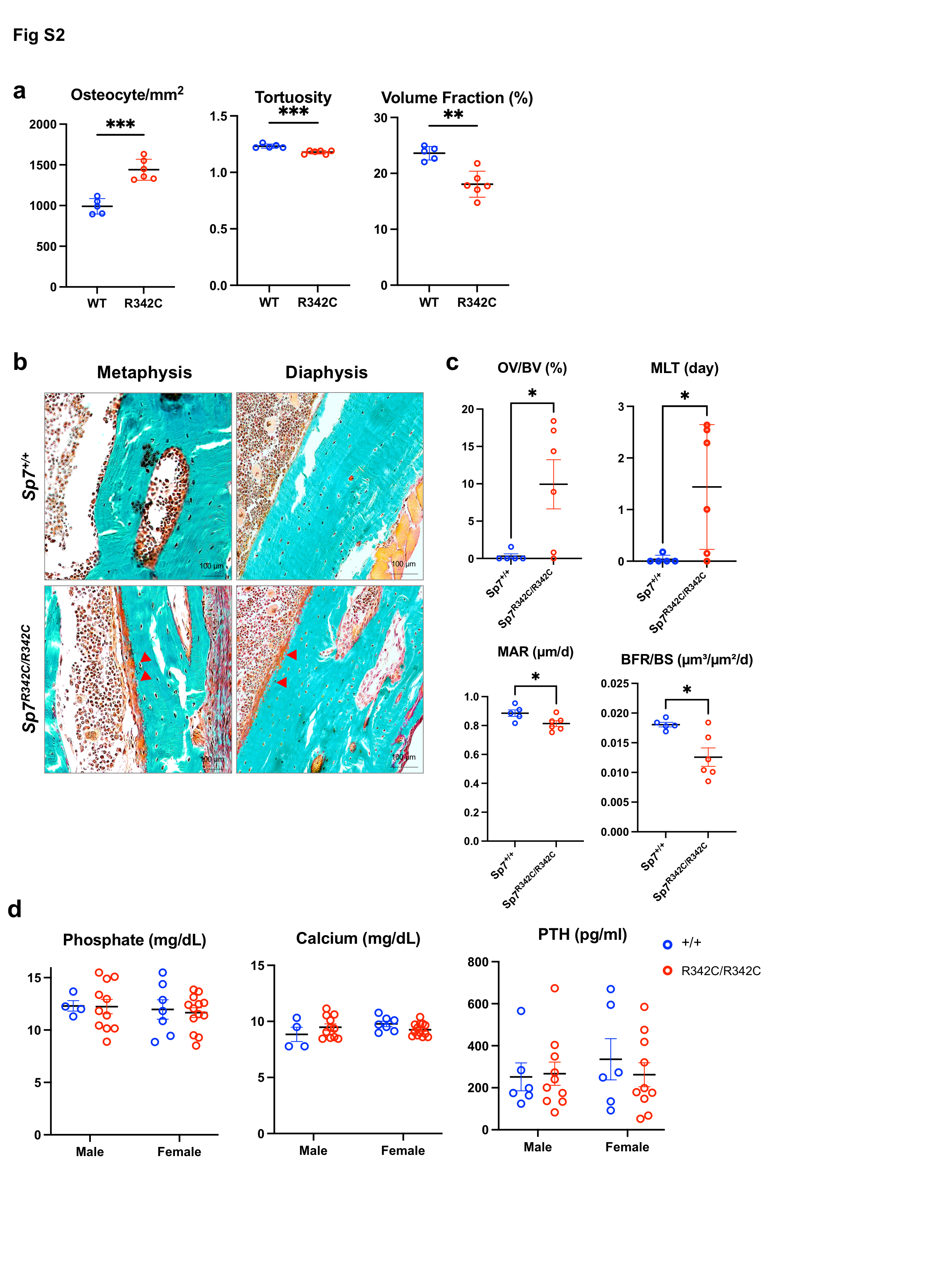

Supplement: Supplementary file 2 — Sup Figure 2 [file 41413_2025_440_MOESM2_ESM.tif]

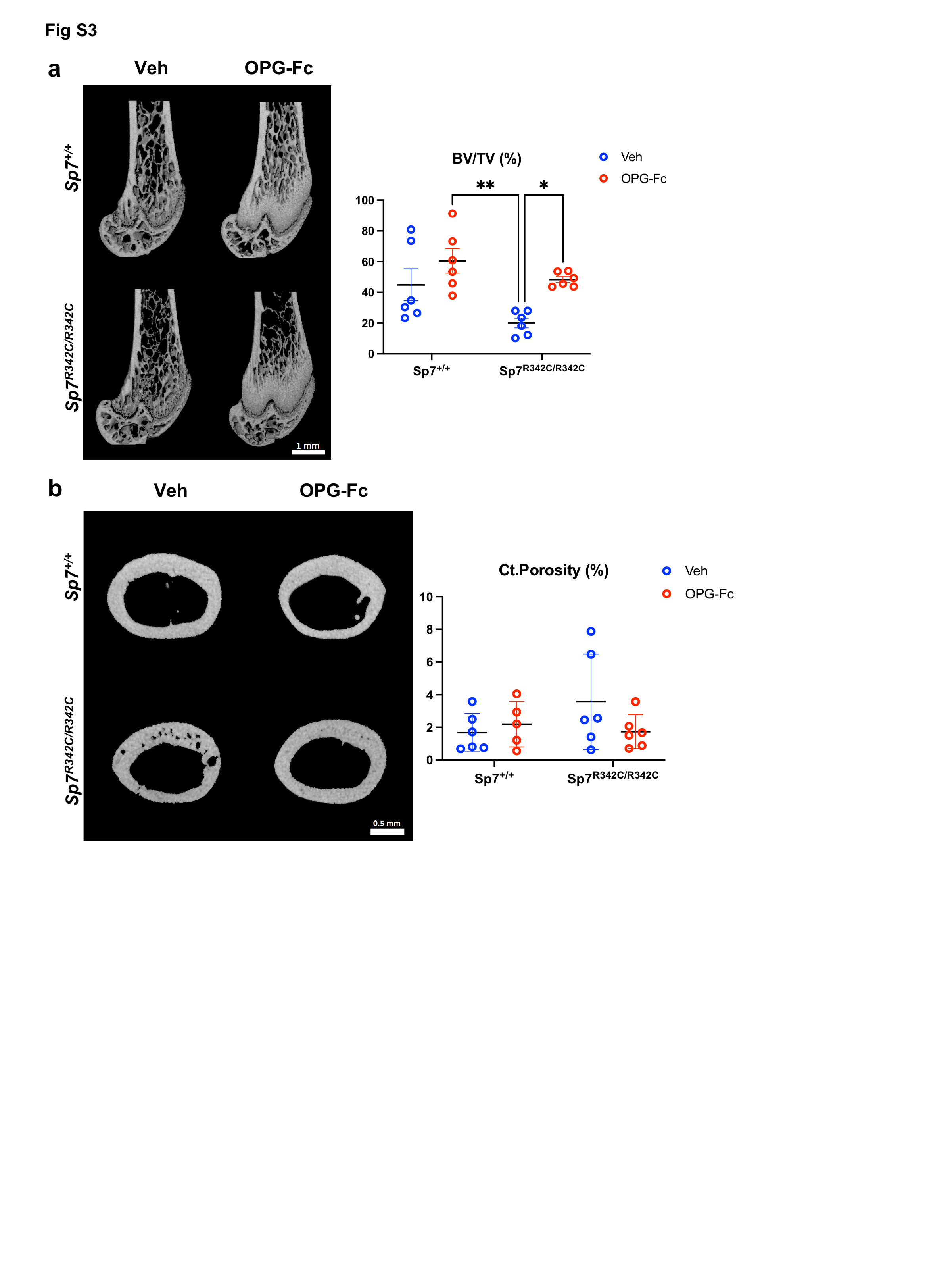

Supplement: Supplementary file 3 — Sup Figure 3 [file 41413_2025_440_MOESM3_ESM.tif]
