# Supplementary material for: Osteoclast-independent osteocyte dendrite defects in mice bearing the osteogenesis imperfecta-causing Sp7 R342C mutation
Source: Bone Res. 2025 Jul 19;13:70. doi: 10.1038/s41413-025-00440-1 (PMC12276330; doi:10.1038/s41413-025-00440-1)
Supplement: Supplementary file 10 — Supplementary legends [file 41413_2025_440_MOESM10_ESM.docx]

**Fig S1. Increased ductility in femurs of *Sp7^R342C^* knock-in mice. a-b** Three-point bending experiment was performed in the right femur of 10-week-old female mice. Student’s *t* test was performed for statistical analysis, and *P* values are shown (**P* < 0.05; ***P* < 0.01, ****P* < 0.001, *****P* < 0.0001).

**Fig S2. Increased osteoid and reduced mineralization in *Sp7^R342C^* mutant mice. a** Quantification of osteocyte density, tortuosity, and the lacunar-canalicular network (LCN) volume fraction in 3D. **b** Femurs from 8-week-old mice have been subjected to bone histomorphometric analysis. Trichrome staining shows increased osteoid in *Sp7^R342C/R342C^* mice (red arrowheads) in both metaphysis and diaphysis. **c** Quantification of osteoid volume (OV/BV), mineralization lag time (MLT), mineral apposition rate (MAR) and bone formation rate (BFR/BS) in the metaphysis. **d** Serum PTH, phosphate and calcium levels are not affected in *Sp7^R342C/R342C^* mice compared to controls. Student’s *t* test was performed for statistical analysis in **a** and **c** and two-way ANOVA analysis followed by post hoc Tukey–Kramer test was performed in **d** (**P* < 0.05; ***P* < 0.01, ****P* < 0.001, *****P* < 0.0001). Scale bars are shown in the images.

**Fig S3. OPG-Fc treatment increases trabecular bone mass.** **a** μCT images from the femoral metaphysis of 10-week-old female mice. Trabecular bone mass increases in the OPG-Fc treated group compared to the vehicle-treated group. **b** μCT images from the femoral diaphysis of 10-week-old female mice. Cortical porosity is partially reduced in the OPG-Fc treated group compared to the vehicle-treated group. Two-way ANOVA analysis followed by post hoc Tukey–Kramer test was performed (**P* < 0.05; ***P* < 0.01, ****P* < 0.001, *****P* < 0.0001). Scale bars are shown in the images. All images in the same panel have the same scale.
